# Long non-coding RNA ATB is associated with metastases and promotes cell invasion in colorectal cancer via sponging miR-141-3p

**DOI:** 10.3892/etm.2022.11163

**Published:** 2022-01-24

**Authors:** Xianming Liu, Cunchuan Wang

Exp Ther Med 20:Article no. 261, 2020; DOI: 10.3892/etm.2020.9391

Following the publication of the above article, the authors have realized that they inadvertently included data derived from the same original source for the invasion and migration assay Vector control experiments shown for the HCT116 cells in Fig. 3C on p. 7. After having re-examined their original data, the authors have realized that the data for the Invasion assay experiment in Fig. 3C were chosen incorrectly.

The corrected version of Fig. 3, including the correct data panel for the Invasion assay / Vector control experiment for the HCT116 cells in Fig. 3C, is shown on the next page. Note that the error made during the assembly of this figure did not affect the overall conclusions reported in the paper. All the authors agree with the publication of this corrigendum, and are grateful to the Editor of *Experimental and Therapeutic Medicine* for allowing them the opportunity to publish this. They also apologize to the readership for any inconvenience caused.

## Figures and Tables

**Figure 3:**
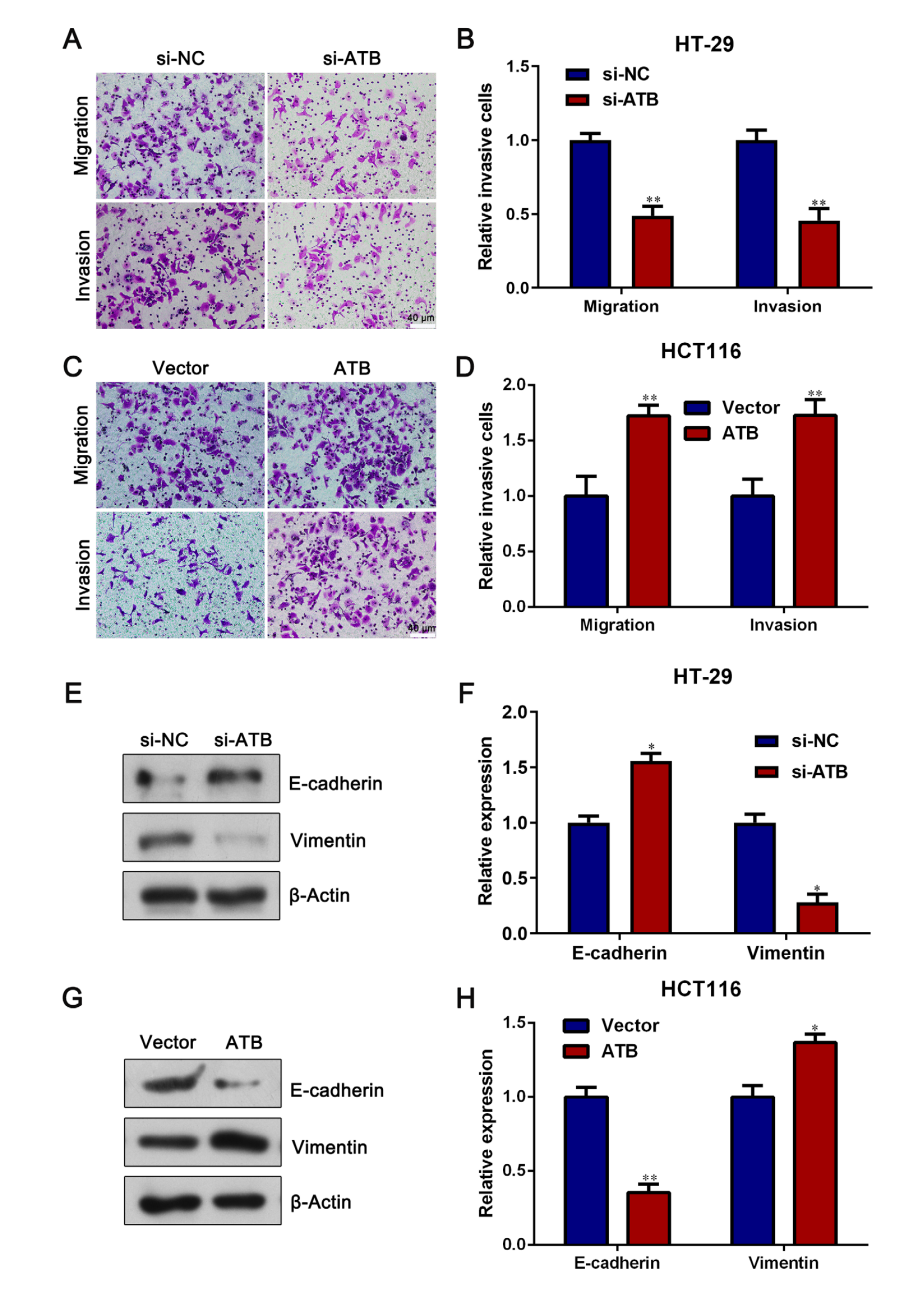
lncRNA-ATB promotes CRC cell migration. (A) Representative images of the Transwell assay in HT-29 cells. (B) Quantification of the effect of lncRNA-ATB knockdown on HT-29 cell migration and invasion. (C) Representative images of the Transwell assay in HCT116 cells. (D) Quantification of the effect of lncRNA-ATB overexpression on HCT116 cell migration and invasion assay. E-cadherin and Vimentin expression levels were (E) determined via western blotting and (F) semi-quantified in lncRNA-ATB-knockdown HT-29 cells. E-cadherin and Vimentin expression levels were (G) determined via western blotting and (H) semi-quantified in lncRNA-ATB-overexpression HCT116 cells. ^*^P<0.05 and ^**^P<0.01 vs. si-NC or Vector. lncRNA-ATB, long non-coding RNA-activated by transforming growth factor-β; CRC, colorectal cancer; NC, negative control; si, small interfering RNA.

